# Engineering thermal stability and solvent tolerance of the soluble quinoprotein PedE from *Pseudomonas putida* KT2440 with a heterologous whole‐cell screening approach

**DOI:** 10.1111/1751-7915.13036

**Published:** 2017-12-14

**Authors:** Matthias Wehrmann, Janosch Klebensberger

**Affiliations:** ^1^ Institute of Technical Biochemistry University of Stuttgart Stuttgart Germany

## Abstract

Due to their ability for direct electron transfer to electrodes, the utilization of rare earth metals as cofactor, and their periplasmic localization, pyrroloquinoline quinone‐dependent alcohol dehydrogenases (PQQ‐ADHs) represent an interesting class of biocatalysts for various biotechnological applications. For most biocatalysts protein stability is crucial, either to increase the performance of the protein under a given process condition or to maximize robustness of the protein towards mutational manipulations, which are often needed to enhance or introduce a functionality of interest. In this study, we describe a whole‐cell screening assay, suitable for probing PQQ‐ADH activities in *Escherichia coli* BL21(DE3) cells, and use this assay to screen smart mutant libraries for increased thermal stability of the PQQ‐ADH PedE (PP_2674) from *Pseudomonas putida* KT2440. Upon three consecutive rounds of screening, we identified three different amino acid positions, which significantly improve enzyme stability. The subsequent combination of the beneficial mutations finally results in the triple mutant R91D/E408P/N410K, which not only exhibits a 7°C increase in thermal stability but also a twofold increase in residual activity upon incubation with up to 50% dimethyl sulfoxide (DMSO), while showing no significant difference in enzymatic efficiency (*k*_cat_/*K*_M_).

## Introduction

Dehydrogenases are important industrial enzymes with biotechnological applications ranging from the selective oxidation of alcohols and aldehydes to the regioselective oxidation of polyols, the production of enantiopure alcohols *via* kinetic resolution or the oxidation of prochiral compounds (Patel, 2004; Liese and Seelbach, 2006; Yakushi and Matsushita, 2010; Hollmann *et al*., 2011; Romano *et al*., 2012). Among alcohol dehydrogenases (ADHs), the pyrroloquinoline quinone (PQQ)‐dependent ADHs are of particular interest for several reasons. First, they can be coupled to electrodes, rendering them suitable for biosensor and fuel‐cell applications (Igarashi *et al*., 2004; Treu and Minteer, 2008; Takeda *et al*., 2013; Scherbahn *et al*., 2014; Guo *et al*., 2016). Second, these enzymes are localized within the periplasm of Gram‐negative cells, thus eliminating the need for energy‐driven transport of target chemicals into the cytoplasm. Together with the irreversible nature of the catalyzed reaction, this leads to unhindered accumulation of oxidized products outside of the cells. For living biocatalysts, this can be of great advantage as it minimizes toxic effects of substrates and corresponding oxidation products (Acharya and Manning, 1983; Huang *et al*., 2005). Third, PQQ‐dependent enzymes do not interfere with the intracellular NAD^+^/NADH and NADP^+^/NADPH homeostasis, which is beneficial to maintain metabolic functionality of the biocatalyst (Matsushita *et al*., 2002; Chen *et al*., 2014). Last, the recent identification of specific PQQ‐ADHs which utilize lanthanides as metal cofactor has fuelled the idea of using these enzymes or the corresponding bacterial hosts in biomining and recycling processes of rare earth metals (Skovran and Martinez‐Gomez, 2015; Martinez‐Gomez *et al*., 2016; Wehrmann *et al*., 2017).

For many biocatalytic applications, enzyme stability is a key criterion (Polizzi *et al*., 2007; Woodley, 2013). This is particularly true for enzymes located in the periplasmic space or enzymes used as purified entities outside of the protective context of the cell. Additionally, enzyme stability has also been associated with increased evolvability and mutational robustness, which is important for the development of biocatalysts with new and/or specific functionalities (Bloom *et al*., 2006; Bloom and Arnold, 2009; Tokuriki and Tawfik, 2009).

As a first step towards establishing soluble PQQ‐ADHs as novel tools for biocatalysis, the main objective of this study was hence to increase the stability of our model quinoprotein PedE (PP_2674) from *Pseudomonas putida* KT2440. Different methods have been described to rationally engineer increased stability of proteins. These include the introduction of disulphide bonds (Yin *et al*., 2015), identifying mutations based on free energy calculations (Schymkowitz *et al*., 2005; Kellogg *et al*., 2011), protein surface charge optimization (Gribenko *et al*., 2009), B‐factor analysis (Reetz *et al*., 2006), homologous comparison (Lehmann *et al*., 2000) and combinations of the before‐mentioned methods (Jochens *et al*., 2010; Cerdobbel *et al*., 2011; Blum *et al*., 2012; Wijma *et al*., 2014).

To engineer proteins for a desired function, suitable whole‐cell assays are highly beneficial, as they allow efficient screening of mutant libraries without prior purification of the different protein variants. Notably, two recent studies reported whole‐cell assays, allowing activity screens of native membrane‐associated PQQ‐ADHs using *Gluconobacter* and *Methylobacterium* strains (Peters *et al*., 2013; Vemuluri *et al*., 2017). However, to the best of our knowledge, a heterologous system suitable for efficient production, purification and engineering of soluble PQQ‐ADHs has not been developed thus far.

With this study, we describe the engineering of the calcium‐dependent quinoprotein PedE from *P. putida* KT2440 towards increased thermal stability. For this, a heterologous whole‐cell screening assay based on the well‐characterized protein expression host *Escherichia coli* BL21(DE3) was developed, which allows the functional expression, purification and comparable quantification of enzyme activities for different quinoproteins. Increased stability was achieved using a combinatorial strategy targeting flexible residues with smart libraries of naturally occurring amino acids at the corresponding positions. With this approach, several beneficial mutations were identified and, together with combinatorial variants thereof, biochemically characterized for their stability towards elevated temperatures and the presence of the model organic solvent dimethyl sulfoxide (DMSO).

## Results

### Characterization of soluble quinoproteins in cells of E. coli BL21(DE3)

Aim of this study was to engineer increased stability of the PQQ‐ADH PedE (PP_2674) from *P. putida* KT2440, a model organism for biocatalysis and environmental engineering approaches (Poblete‐Castro *et al*., 2012; Nikel *et al*., 2014, 2016; Loeschcke and Thies, 2015). To achieve this, we initially started to establish an engineering platform for soluble PQQ‐ADHs based on the heterologous production host *E. coli* BL21(DE3). In particular, a recently described DCPIP‐dependent colorimetric assay (Peters *et al*., 2013) was adapted for the quantification of enzymatic activities of heterologously produced PQQ‐ADHs in living cells of *E. coli* (see Fig. S3 and Table S2). To test the broad applicability of the adapted assay, calcium (Ca^2+^)‐ and lanthanide (Ln^3+^)‐dependent PQQ‐ADHs from different organisms, namely PedE (Ca^2+^) and PedH (PP_2679; Ln^3+^) of *P. putida* KT2440 (Takeda *et al*., 2013; Wehrmann *et al*., 2017) as well as ExaA (PA1982; Ca^2+^) of *Pseudomonas aeruginosa* (Rupp and Görisch, 1988; Chattopadhyay *et al*., 2010), were expressed in *E. coli* BL21(DE3) cells and activities for all enzymes were determined.

**Figure 1:**
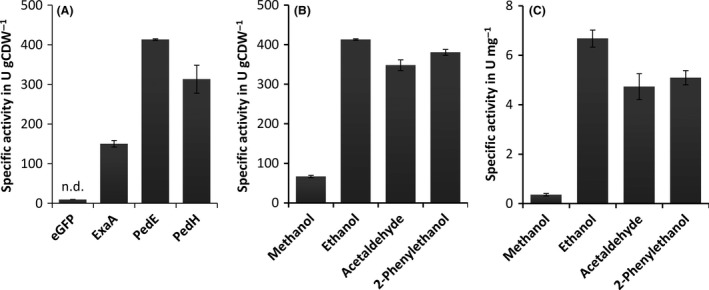
(A) Specific enzyme activities of the Ca^2+^‐dependent pyrroloquinoline quinone‐dependent alcohol dehydrogenases (PQQ‐ADHs ExaA of *Pseudomonas aeruginosa* and PedE of *Pseudomonas putida* KT2440 as well as the lanthanide‐dependent PQQ‐ADH PedH of *P. putida* KT2440 determined using an *E. coli* BL21(DE3) based whole‐cell assay with 10 mM ethanol as substrate. (B) Specific activities of PedE determined with an *E. coli* BL21(DE3) based whole‐cell assay normalized to cell dry weight with different substrates at a concentration of 10 mM. (C) Specific enzymatic activity of purified PedE protein with different substrates at a concentration of 10 mM. Data are presented as the mean value of three replicates and error bars represent the corresponding standard deviation. Activities below detection limit are indicated (n.d.).

With these experiments, we found activities for all tested PQQ‐ADHs when 10 mM of ethanol was used as substrate (Fig. 1A). In contrast, control cells producing eGFP from the same expression vector showed no detectable enzyme activity. To further demonstrate the robustness and versatility of the whole‐cell assay, PedE activities on four different substrates, namely methanol, ethanol, 2‐phenylethanol and acetaldehyde, were determined (Fig. 1B) and compared with activities obtained with the purified enzyme (Fig. 1C). Under both conditions, the highest specific activity was found with ethanol as substrate (413 ± 2 U gCDW^−1^ and 6.7 ± 0.3 U mg^−1^). When using purified enzyme, 2‐phenylethanol was converted with 76 ± 6% followed by acetaldehyde with 71 ± 9% of the activity determined with ethanol. For methanol, the activity was less than a tenth of that determined for ethanol (5 ± 1%). Notably, a similar trend in enzyme activities with all substrates was observed when using the whole‐cell assay, however, with slightly higher relative activities, namely 92 ± 2%, 84 ± 3% and 16 ± 1% for 2‐phenylethanol, acetaldehyde and methanol, respectively. To show that assay activity measurements correspond to product formation, additional biotransformations with PedE were carried out in a whole‐cell set‐up and with the purified enzyme. These experiments revealed that phenylacetaldehyde is the main oxidation product of 2‐phenylethanol in both experimental set‐ups and that product formation only occurred in the presence of the electron mediator PMS and the terminal electron acceptor DCPIP (Table S3).

### Identification of amino acid residues in PedE to increase thermal stability

To engineer increased thermal stability of the quinoprotein PedE, we aimed for a combination of the B‐FITTER approach and the consensus method (Lehmann *et al*., 2000; Reetz *et al*., 2006), similar to a recent study by Jochens *et al*. (2010). The B‐FITTER strategy uses the concept that temperature‐mediated unfolding of protein structures (denaturation) is initiated at flexible residues within the protein scaffold and thus, rigidifying those positions can improve the stability of an enzyme (Reetz *et al*., 2006, 2010; Yu and Huang, 2014). Consequently, we initially determined the most flexible residues within PedE. As no crystal structure of PedE was available, the B‐values from the crystal structure of the PQQ‐ADH ExaA of *P. aeruginosa* (sequence identity 88%; PDB entry: 1FLG) were derived using the b‐fitter tool (Reetz *et al*., 2006). The corresponding positions of ExaA in PedE were identified through a 3DM database consisting of about 12,000 sequences showing structural similarity to PedE (Kuipers *et al*., 2010). Of the residues with the highest B‐values that were included in the 3DM database, residues D307, N310, E408, K352, N410 and R91 were chosen for mutagenesis (Table 1). In a second step, the most frequently occurring amino acids at these positions were selected to create small focused libraries from two subsets of the 3DM database that contained only sequences encoding for PQQ‐dependent ADHs (1315 sequences) or sequences from extremophile organisms (111 sequences; see Table 1). Finally, a minimal degenerated codon, which allowed the substitution for all variants of interest, was used to generate five mutant libraries containing between five and ten variants.

**Table 1:** Composition of the small focused mutant libraries used in the study. *Black dots* mark the most abundant amino acids at the corresponding position in a subset of the 3DM database that only contained pyrroloquinoline quinone‐dependent alcohol dehydrogenases (PQQ‐ADHs). *Red dots* mark the amino acids frequently present in a subset of the 3DM database that contained only sequences of proteins derived from extremophile organisms

Position	WT sequence	B‐Factor	Library composition	Degenerate codons	Screened clones
91	R	39	A•, D  , Q•, E•, K•	VAG, GSC	36
307	D	63	E•, I•, K  , T  , V•	RHG, ATC	30
310	N	63	R•, E•, Q•, K•, T•	VMG, CGC	30
352	K	41	A•, R  , D•, Q•, W 	GMM, CGC, TGG	42
408	Q	46	G•, K•, M•, P•, Y 	MHG, KRC	84
410	N	39	A•, Q  , K  , S•, T•	NCG, RAM	25


 = PQQ‐ADH data set; • = Extremophile data set.

### Screening of mutant libraries and identification of beneficial combinatorial mutations

**Figure 2:**
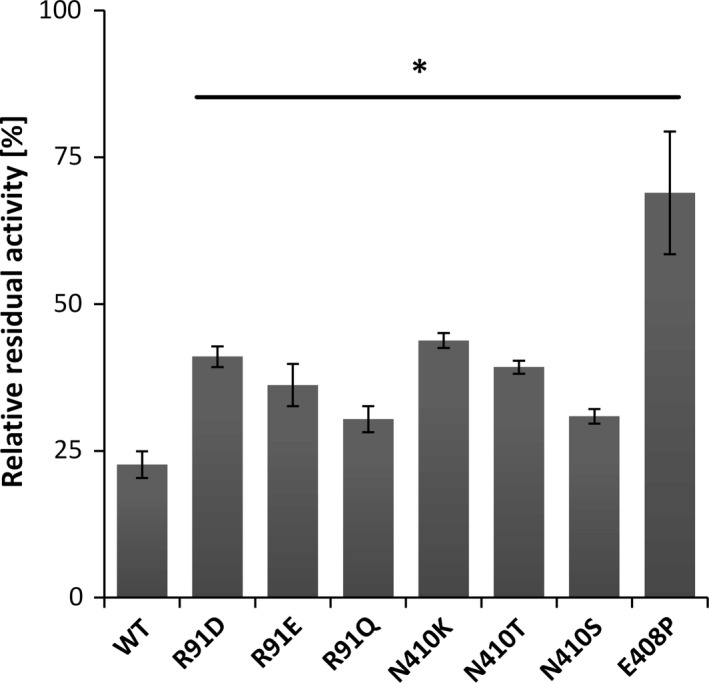
Relative residual activity upon 1 h incubation at 45°C of identified thermostable single mutants. The residual activity of each mutant was statistically analysed (two‐tailed *t*‐test; *α *= 0.05; *N *=* *5; graphpad prism, version 7.03) and found to be significantly different from the wild‐type allele (*; p < 0.01). Data are presented as mean values of biological triplicates, and error bars represent the corresponding standard deviations.

A total of about 200 clones were screened for increased thermal stability using the heterologous whole‐cell assay to guarantee 95% coverage of each mutant library. Upon three consecutive rounds of screening (see Material and Methods for details), seven variants from three different libraries were identified, which exhibited significantly increased thermal stability (two‐tailed *t*‐test, *N *=* *5, *α *= 0.05; graphpad prism, version 7.03) upon 1 h incubation at 45°C (relative residual activity; Fig. 2). After sequence analysis, different amino acid substitutions at three positions were found. Three variants were identified at positions R91 (D, Q, E) and N410 (K, S, T) while only one variant was found at position E408 (P). The best mutants from each library were R91D, N410K and E408P with 41 ± 1.7%, 44 ± 1.3% and 69 ± 10% relative residual activities compared with 23 ± 2.3% for the wild‐type allele. Notably, two of the three variants, namely R91D and N410K, were derived from the 3DM database subset containing only sequences from extremophile organisms. Furthermore, no variants with increased thermal stability at positions 307, 310 and 352 were identified.

**Figure 3:**
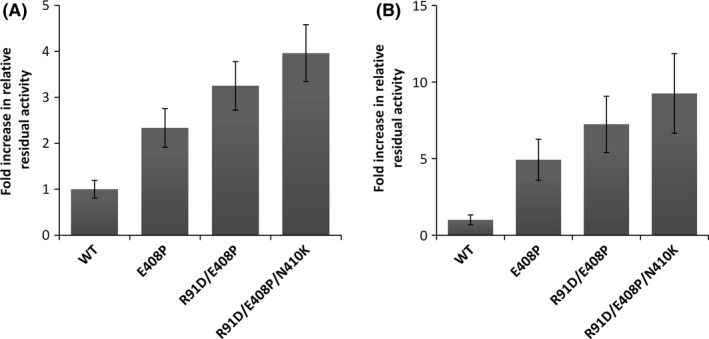
Fold increase in relative residual activity upon 1 h incubation at 45°C of stability mutants compared with PedE wildtype determined using *Escherichia coli* BL21(DE3) whole cells (A) or at 65°C for purified proteins (B). Data are presented as the mean of three biological triplicates and error bars represent the corresponding standard deviations.

To test whether a combination of the mutations would result in additional stability, the most thermostable variations were combined using mutant E408P as starting point. Data obtained with the whole‐cell activity assay showed that each consecutive mutation had an additive effect. While the best single mutant E408P exhibited a 2.3 ± 0.4‐fold increased thermal stability, the R91D/E408P double mutant showed a 3.2 ± 0.5 and the R91D/E408P/N410K triple mutant a 4.0 ± 0.6‐fold increased stability upon incubation at 45°C for 1 h compared with the wild‐type allele (Fig. 3A).

### Characterization of thermostable alleles as purified enzymes

**Figure 4:**
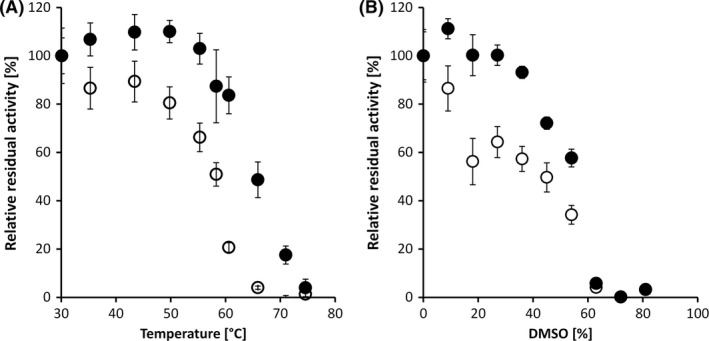
(A) Relative residual activities of the purified PedE wild‐type enzyme and the R91D/E408P/N410K mutant allele upon 1 h incubation at various temperatures. (B) Residual activity of purified PedE wild‐type enzyme and the R91D/E408P/N410K mutant allele upon 1 h incubation with various concentrations of the organic solvent DMSO. Data are presented as the mean of three biological triplicates and error bars represent the corresponding standard deviations.

To verify the increased thermal stability of the PedE alleles, the C‐terminally His‐tagged single, double and triple mutants as well as the wild‐type enzyme were purified by immobilized‐metal affinity chromatography to visual homogeneity (Fig. S1). As the *apo* forms of the enzymes did not show any residual activity upon incubation at 45°C for 1 h (Fig. S2), a reconstitution step with PQQ and CaCl_2_ was performed prior to the experiments. Interestingly, the reconstituted wild‐type enzyme showed much higher residual activities upon incubation at 45°C for 1 h (87 ± 6%, Fig. S2) compared with the values determined for this enzyme in the whole‐cell assay (23 ± 2%, Fig. 2). However, when the incubation temperature for the purified enzyme was increased to 65°C, a similar trend as in the whole‐cell assay was observed. Under these conditions, steadily increasing thermal stabilities starting from the E408P single mutant (4.9 ± 1.3‐fold), over the R91D/E408P double mutant (7.2 ± 1.8‐fold), up to the R91D/E408P/N410K triple mutant (9.3 ± 2.6‐fold) were observed (Fig. 3B). By determining the *T*_50_^60^ values, representing the temperature required to reduce the initial activity of the enzyme to 50% upon 1 h of incubation, we revealed that for the R91D/E408P/N410K variant this value was increased by 7°C from 58°C to 65°C compared with the wild‐type allele (Fig. 4A). In addition to the increased thermal stability, we further found that the R91D/E408P/N410K variant exhibited an increased stability towards the organic solvent dimethyl sulfoxide (DMSO), reflected by an about twofold increased relative residual activity after incubation with 20–50% DMSO for 1 h compared with the wild‐type enzyme (Fig. 4B). However, no enzyme activity was detectable with PedE or the R91D/E408P/N410K variant upon incubation with more than 60% DMSO.

Finally, kinetic parameters of the wild‐type enzyme and the R91D/E408P/N410K variant were determined with the model substrate ethanol. We found that although the maximum velocity *V*_max_ of the wild‐type enzyme was significantly (two‐tailed *t*‐test; *α *= 0.05; *N *=* *3; graphpad prism, version 7.03) higher than that of the triple mutant (5.0 ± 0.2 U mg^−1^ vs 6.1 ± 0.2 U mg^−1^), no significant differences in the substrate affinities (*K*_M_) or enzymatic efficiencies (*k*_cat_/*K*_M_) were detected (Table 2).

**Table 2:** Kinetic constants of the PedE wildtype and the R91D/E408P/N410K triple mutant with ethanol as substrate. The maximum velocities (*V*_max_), substrate affinities (*K*_M_) and catalytic efficiencies (*k*_cat_/*K*_M_, were *k*_cat_ is the turnover frequency per cofactor molecule) were derived from non‐linear regression of specific activities to a Michaelis–Menten model. Kinetic constants are presented as best‐fit values ± the standard errors. Significant differences between the enzymes are marked (*, two‐tailed *t*‐test, *α *= 0.05, *N *=* *3; graphpad prism, version 7.03)

Enzyme	*V*_max_ [U mg^−1^]	*K*_M_ [μM]	*k*_cat_/*K*_M_ [mM s^−1^]
WT	6.1 ± 0.2*	74 ± 11	93 ± 15
R91D/E408P/N410K	5.0 ± 0.2*	61 ± 14	92 ± 21

## Discussion

Screening mutant libraries remains one of the major bottlenecks in most protein engineering approaches. Thus, a robust assay for rapid activity screenings in whole cells based on the protein expression strain *E. coli* BL21(DE3) was developed. After optimization of the assay conditions, the relative activities determined for the PedE enzyme with four model substrates were found to be very similar to those determined with the purified enzyme. It was further demonstrated that the whole‐cell assay allows robust and comparable quantification of enzyme activities of PQQ‐ADH enzymes from various organisms and with different cofactor dependencies. Together with the advantages of working with the laboratory workhorse *E. coli* BL21(DE3) as a screening host, permitting the use of standard protocols for efficient and effortless production and purification of plasmids and enzymes, these results show that the developed assay represents a suitable tool for rapid quantification of enzyme activities without the prior need for labour intensive and time‐consuming cell disruption and protein purification procedures.

For subsequent stability engineering, a combination of the b‐fitter and the consensus approach was used to generate small focused libraries consisting of the most frequently occurring amino acids at six positions of high flexibility (Lehmann *et al*., 2000; Reetz *et al*., 2006).

**Figure 5:**
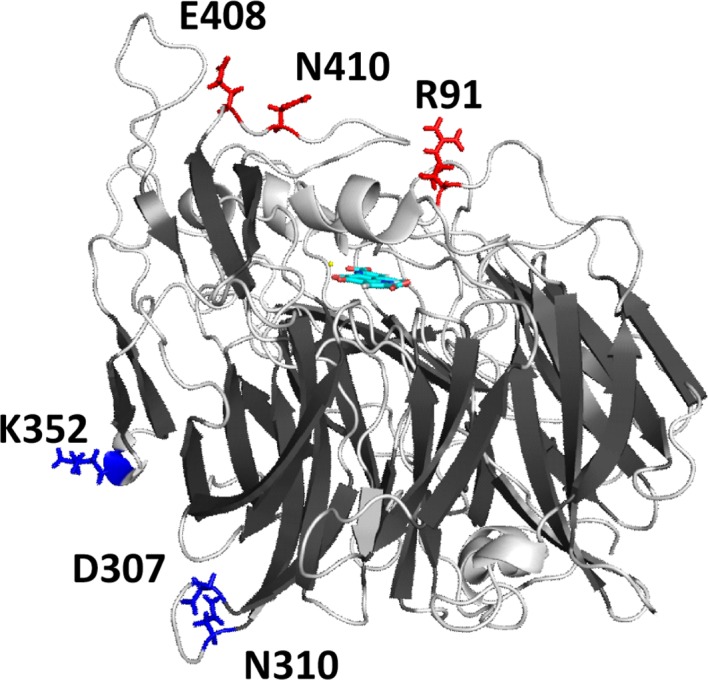
Cartoon representation of a homology model of PedE with residues 91, 408 and 410 pictured as red sticks and residues 307, 310 and 352 pictured as blue sticks. Ca^2+^ ion in the active site is represented as a yellow sphere, and the PQQ cofactor is represented as a stick model with an element colour code (C, cyan; O, red; N, blue).

From screening those libraries with the developed assay, we found that the introduction of conserved residues at the positions 91, 408 and 410 resulted in improved thermal stability of the enzyme. Notably, these residues are all within 20 Å of the PQQ cofactor‐binding site and on the same side of the β‐barrel structure of the enzyme as the active site (Fig. 5). In contrast, positions 307, 310 and 352, for which no variants with improved thermal stability could be identified, are further distant from the active site and on the other side of the β‐barrel structure. This suggests that rigidifying flexible residues close to the active site is more beneficial compared with more distant residues. A similar effect has also been reported for the lipases CalB *of Candida antarctica* and Lip1 of *Candida rugosa* (Xie *et al*., 2014; Zhang *et al*., 2016). The two studies suggest that for large proteins, mutating flexible residues located far from the active site, does not protect the active centre from heat‐induced detrimental conformational changes. In this context, it is further worthwhile noting that we found that the purified *apo*‐form of PedE is much less thermostable compared with the PQQ reconstituted form of PedE. This strongly indicates that binding of the cofactor to the active site is not only essential for the catalytic mechanism but also plays a crucial role in stabilization of the enzyme.

As described for many other enzymes, the combination of beneficial mutations had an additive effect and finally led to the PedE variant R91D/E408P/N410K, which exhibited an about 10‐fold improved thermal stability upon incubation at 65°C and a 7°C increased T_50_^60^ of 65°C compared to 58°C for the wild‐type allele. In addition, the R91D/E408P/N410K variant showed an improved stability in the presence of the hostile organic solvent DMSO. A similar correlation between temperature stability and solvent tolerance has also been reported for several other enzymes (Cowan, 1997; Hao and Berry, 2004; Reetz *et al*., 2010). Despite the fact that the underlying molecular mechanism of this correlation is not well understood, it is of particular interest for many biotechnological applications, where solvent tolerance is a key factor (Schmid *et al*., 2001; Bornscheuer *et al*., 2012).

Comparison of kinetic parameters revealed that while a slight decrease in reaction velocity (*V*_max_) from 6.1 to 5.0 U mg^−1^ was observed for the R91D/E408P/N410K variant when compared with the wild‐type allele, no significant differences were found with respect to the substrate affinity (*K*_M_) or enzymatic efficiencies (*k*_cat_/*K*_M_). Hence, a dramatically more thermostable and solvent‐tolerant mutant with only minor impairment on the enzyme kinetics at 30°C has been generated. However, as no screening for increased activity at 30°C was performed, it is very likely that by performing additional rounds of directed evolution, mutations that compensate for the decreased *V*_max_ can be identified.

Overall, in this study, we describe a heterologous whole‐cell screening assay for enzyme activity measurements of soluble quinoproteins, and its subsequent use to identify different variants of the PQQ‐ADH PedE from *P. putida* KT2440 with increased thermal stability. We further provide new insights into the impact of the quinone cofactor on the stability of PQQ‐dependent enzymes and identify the combinatorial variant R91D/E408P/N410K, which shows significantly improved thermal stability and tolerance towards the organic solvent DMSO, and can now serve as a starting point for future applications including the engineering towards novel enzymatic functions.

## Experimental procedures

### Strains and growth conditions

*Escherichia coli* BL21(DE3) was used as host for cloning and expression. If not stated otherwise, cells were routinely cultured at 37°C in liquid LB medium (Maniatis *et al*., 1982) on a rotary shaker at 180 rpm (HT Minitron, Infors AG, Einsbach, Germany). For plasmid maintenance and selection, 40 μg ml^−1^ kanamycin or 100 μg ml^−1^ ampicillin was supplemented to the medium.

### Plasmid construction, library generation and evaluation

A detailed description of the construction of the broad‐host‐range expression vectors pJoe4036.1::*exaA*(His)6, pMW09 and pMW10 containing the *exaA* gene (GI: 15597178) from *P. aeruginosa*,* pedE* gene (GI: 26989393) and *pedH* gene (GI: 26989398) both from *P. putida* KT2440 with a C‐terminal 6xHis‐Tag that were used in this study can be found elsewhere (Chattopadhyay *et al*., 2010; Wehrmann *et al*., 2017). Small focused mutant libraries were generated using multichange isothermal mutagenesis (Mitchell *et al*., 2013). For this, the minimal degenerate codon combinations for the desired variance at each position was determined using MDC analyzer (Tang *et al*., 2014). For mutagenesis, self‐complementary primers containing the desired degenerate codon in its centre, as well as primers that contained homologies to the plasmid pMWH09, were designed (see Table S1). Upon PCR, the two fragments and the HindIII and NdeI digested vector were assembled in a one‐step isothermal assembly reaction, diluted threefold in MilliQ H_2_O and transformed into electrocompetent *E. coli* BL21(DE3) cells (Gibson, 2011). To verify the desired diversity of the constructed small focused libraries, colonies of one plate from each transformed assembly reaction were resuspended in 2 ml MilliQ H_2_O with a Drigalski spatula, used for a plasmid extraction and submitted for sequence analysis. If the peak height distribution for the corresponding degenerate codon did not match the expectation (Acevedo‐Rocha *et al*., 2015), potentially missing variants were either generated individually or the oversampling number was increased accordingly.

### Whole‐cell activity assay

For enzyme activity measurements of the different PQQ‐ADHs, and variants thereof, a heterologous whole‐cell assay based on *E. coli* BL21(DE3) was developed. For this, a previously described assay in *Gluconobacter oxydans* 621H was used as a starting point (Peters *et al*., 2013) and various parameters including amine source, buffer composition, PQQ, CaCl_2_ or PrCl_3_ supplementation, pH and cell density were optimized (see Table S2 and Fig. S3). In the final protocol, *E. coli* BL21(DE3) cells expressing the desired PQQ‐ADHs (see below) were washed twice, resuspended in 100 mM Tris buffer (pH 8), and adjusted to an optical density between 1 and 4. The reaction buffer contained in a final volume of 250 μl in microtiter plates (96‐well, PS, F‐bottom; Greiner Bio‐one, Frickenhausen, Germany): cell suspension 25 μl, Tris buffer (pH 8) 100 mM, pyrroloquinoline quinone (PQQ) 1 μM, 2,6‐dichlorophenolindophenol (DCPIP) 150 μM, phenazine methosulfate (PMS) 150 μM, imidazole 25 mM, substrate 10 mM, and depending on whether a calcium‐ or lanthanide‐dependent PQQ‐ADH was tested either 1 mM CaCl_2_ or 1 μM PrCl_3_. Due to substrate‐independent background bleaching, the reaction mixture containing the cell suspension was incubated at 30°C for 30 min prior to substrate addition to allow the adjustment of a stable baseline. Enzyme activity was determined by following the reduction in the colorimetric dye 2,6‐dichlorophenolindophenol (DCPIP, extinction coefficient ε = 24.1 cm^−1^ M^−1^ at pH 8) at 600 nm within the first minute upon substrate addition in a microplate reader (POLARstar Omega, BMG Labtech, Ortenberg, Germany). Activities are displayed as units per gram cell dry weight (U gCDW^−1^), given that 1 l of cells at an OD_600_ of 4 corresponds to 1 g cell dry weight, for better comparability.

### Library screening

For the initial screen of the mutant libraries, one colony was used to inoculate 1 ml LB medium containing 10 mM Tris‐HCl pH 7.5; 1 mM CaCl_2_; 40 μg ml^−1^ Kanamycin; 0.6 μM pyrroloquinoline quinone (PQQ); 0.2% rhamnose in 2 ml 96‐deep‐well plates (Carl Roth, Karlsruhe, Germany) and incubated at 16°C and 180 rpm (HT Minitron, Infors AG, Einsbach, German). After 48 h, cells were harvested by centrifugation (4°C, 10 min, 3000 × g), washed twice with 1 ml Tris buffer (100 mM, pH 8), and finally resuspended in 250 μl Tris buffer (100 mM, pH 8). 25 μl of the cell suspension were subsequently used to determine the initial enzyme activity (EA_I_) using the whole‐cell DCPIP assay with 10 mM ethanol substrate as described above. Another 20 μl cell suspension was diluted 1/10 in Tris buffer (100 mM, pH 8) and used to determine the optical density at 600 nm (OD_600_). In addition, 100 μl of each cell suspension was incubated at 45°C in a thermal cycler (Mastercycler ep, Eppendorf AG, Hamburg, Germany) for 1 h and 25 μl were subsequently used for the quantification of the residual activity (EA_R_) using the whole‐cell DCPIP assay with 10 mM ethanol. From samples with an OD_600_ > 1 which showed a higher relative residual activity at 45°C (EA_R_/EA_I_) than samples of the wild‐type variant, six independent clones were used for a second round of screening and analysed for statistically significant improvements compared with the wild‐type allele (two‐tailed *t*‐test, *α *= 0.05, *N *=* *6, graphpad prism (version 7.03), GraphPad Software, Inc., La Jolla, CA, USA. To exclude host effects, plasmids from variants with significantly increased stability were retransformed into *E. coli* BL21(DE3) and re‐evaluated in a third round of screening for improved stability (two‐tailed *t*‐test, *α *= 0.05, *N *=* *5, graphpad prism (version 7.03), GraphPad Software, Inc., La Jolla, CA, USA).

### Expression, purification and characterization of wild‐type enzymes and stability mutants

C‐terminally His‐tagged PQQ‐ADHs were expressed in *E. coli* BL21(DE3), purified using immobilized‐metal affinity chromatography, and characterized for enzymatic activity with different substrates and substrate concentrations as described elsewhere (Wehrmann *et al*., 2017). As the purified *apo*‐enzyme form of PedE did not show any residual activity after incubation at 45°C for 1 h, the minor changes during protein purification and enzyme stability measurements were made as follows: the buffer of the dialyzed enzyme solution was changed from 50 mM Tris buffer (pH 7.5) to 50 mM Tris buffer (pH 7.5) containing 10 μM PQQ and 10 mM CaCl_2_ to allow reconstitution of the enzyme *holo*‐form. To determine the thermal stability of the enzymes, 100 μl of a 100 μg ml^−1^ enzyme solution were incubated at various temperatures for 1 h and the residual activities were determined using an enzyme concentration of 10 μg ml^−1^ in the reaction buffer with 10 mM ethanol as substrate. The final concentrations of components within the assay solution and the procedure for quantifying enzymatic activities, including the preincubation, were identical for purified enzymes and whole cells. From plotting the relative residual activities against the incubation temperature, the T_50_^60^ values (temperature at which after 60 min incubation 50% residual activity is present) were determined. The stability in organic solvents was tested by incubating the reconstituted enzymes in varying concentrations of DMSO at 30°C for 1 h and subsequently measuring the residual enzyme activity with 10 mM ethanol as substrate as described above.

### Enzyme kinetics measurements

The kinetic constants of the enzymes were determined as described previously (Wehrmann *et al*., 2017).

### Homology model

The homology model of PedE was built using Swiss‐Model (Biasini *et al*., 2014). As a template for model construction, the crystal structure of the PQQ‐ADH ExaA of *P. aeruginosa* (PDB entry 1FLG; 88% sequence identity) was used. Model visualization was performed using pymol (Schrodinger LLC, 2015).

### Biotransformations and metabolite quantification

Biotransformations were carried out in a similar reaction buffer as the whole‐cell activity assay. The experiments were performed in a final volume of 500 μl and contained 100 mM Tris buffer (pH 8), 1 μM PQQ, 150 μM DCPIP, 150 μM PMS, 25 mM imidazole, 8 mM 2‐phenylethanol (substrate) and either 50 μl (OD_600_ of 3) of *E. coli* BL21(DE3) cells producing PedE or 10 μg ml^−1^ purified PedE enzyme. At several time points, samples were taken, filtered (0.2 μm) and acidified by adding two equivalents of a 50 mM H_2_SO_4_ solution. Substrates and products were extracted with one equivalent of ethyl acetate, and the organic phase was subsequently analysed by gas chromatography using a GC‐2010 system (Shimadzu, Kyoto, Japan) equipped with a HP‐1 ms UI column (30 m × 0.25 mm × 0.25 μm; Agilent Technologies, Santa Clara, CA, USA) and flame ionization detection. Chromatography was performed with H_2_ as carrier gas at a linear velocity of 30 cm s^−1^. The temperature programme started from 70°C and increased at a rate of 20°C min^−1^ to 140°C, followed by an increase at a rate of 40°C min^−1^ until 320°C, which was finally held for 2 min. The injected volume was 1 μl with a split ratio of 1:10. The measured retention times for the 2‐phenylethanol, phenylacetaldehyde and phenylacetic acid standards were 3.7, 3.2 and 4.5 min respectively.

## Conflict of interest

The authors further declare that the research was conducted in the absence of any commercial or financial relationships that could be construed as a potential conflict of interest.

## Supporting information


**Fig. S1.** SDS‐PAGE analysis of production of PedE WT and stability mutants.
**Fig. S2.** Relative residual activity of purified PedE wildtype protein upon incubation for 1 h at different temperatures with and without reconstitution with CaCl_2_ and PQQ prior to incubation.
**Fig. S3.** Specific activity of *E. coli* BL21(DE3) cells expressing PedE depending on cell density (OD_600_) used.
**Table S1.** Primers used in this study.
**Table S2.** Overview of parameter optimization for the whole‐cell activity assay using of *E. coli* BL21(DE3) cells expressing PedE.
**Table S3.** Biotransformations (500 µl) with 8 mM 2‐phenylethanol using *E. coli* BL21(DE3) cells producing PedE (A) or 10 µg ml^−1^ of purified PedE enzyme (B) in DCPIP assay solution at 30°C.
