# Supplementary material for: Engineering thermal stability and solvent tolerance of the soluble quinoprotein PedE from Pseudomonas putida KT2440 with a heterologous whole‐cell screening approach
Source: Microb Biotechnol. 2017 Dec 14;11(2):399–408. doi: 10.1111/1751-7915.13036 (PMC5812247; doi:10.1111/1751-7915.13036)
Supplement: Supplementary file 1 — Fig. S1. SDS‐PAGE analysis of production of PedE WT and stability mutants. Fig. S2. Relative residual activity of purified PedE wildtype protein upon incubation for 1 h at different temperatures with and without reconstitution with CaCl2 and PQQ prior to incubation. Fig. S3. Specific activity of E. coli BL21(DE3) cells expressing PedE depending on cell density (OD600) used. Table S1. Primers used in this study. Table S2. Overview of parameter optimization for the whole‐cell activity assay using of E. coli BL21(DE3) cells expressing PedE. Table S3. Biotransformations (500 µl) with 8 mM 2‐phenylethanol using E. coli BL21(DE3) cells producing PedE (A) or 10 µg ml−1 of purified PedE enzyme (B) in DCPIP assay solution at 30°C. [file MBT2-11-399-s001.docx]

**Supporting Information**

**Engineering thermal stability and solvent tolerance of the soluble quinoprotein PedE from *Pseudomonas putida* KT2440 with a heterologous whole-cell screening approach**

Matthias Wehrmann and Janosch Klebensberger#

*University of Stuttgart, Institute of Technical Biochemistry, Stuttgart, Germany*

#Correspondence: [janosch.klebensberger@itb.uni-stuttgart.de](mailto:janosch.klebensberger@itb.uni-stuttgart.de)

**
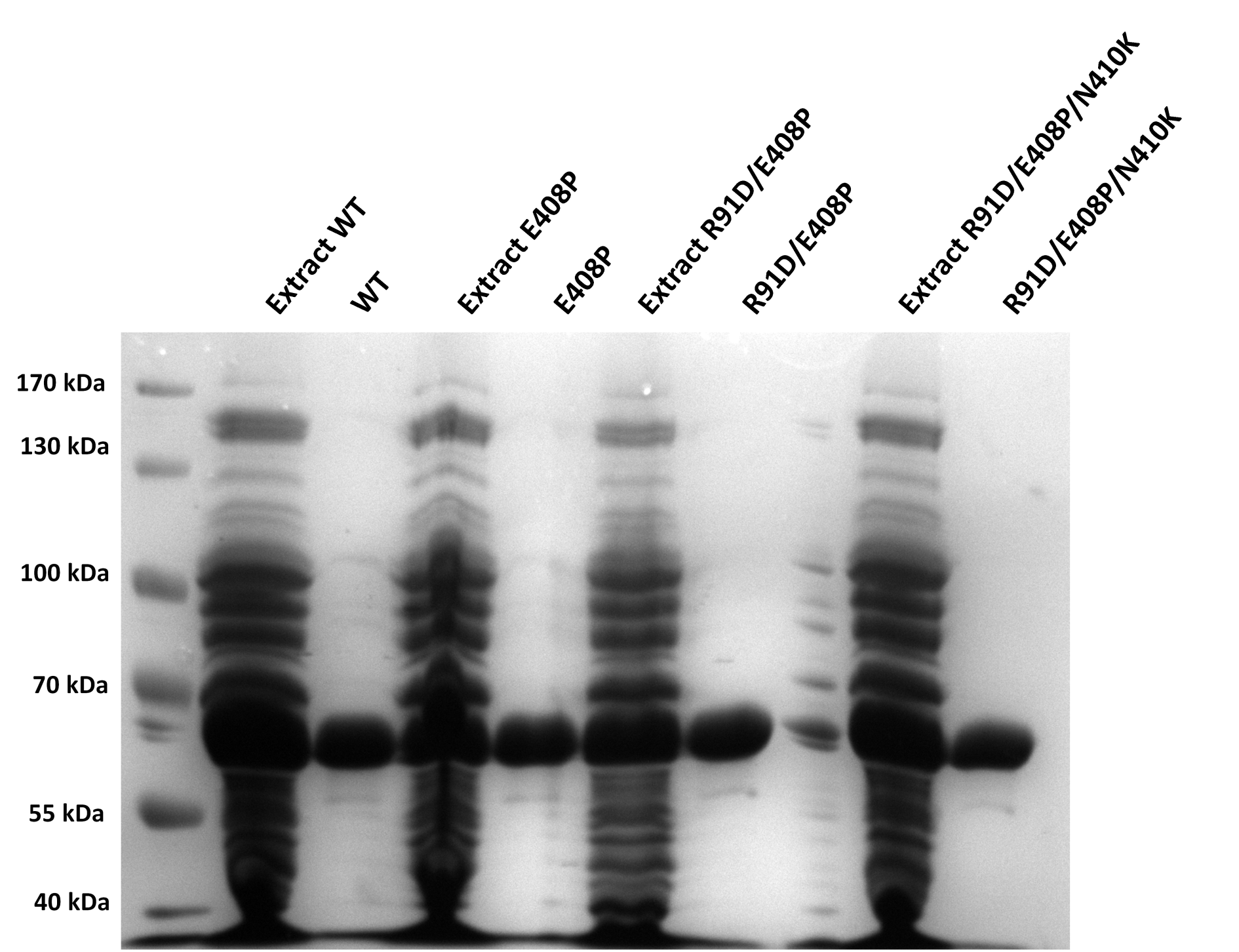
**

**Figure S1:** SDS-PAGE analysis of production of PedE WT and stability mutants. 10 µl cell extract or 20 µg of purified protein were loaded per lane.

**
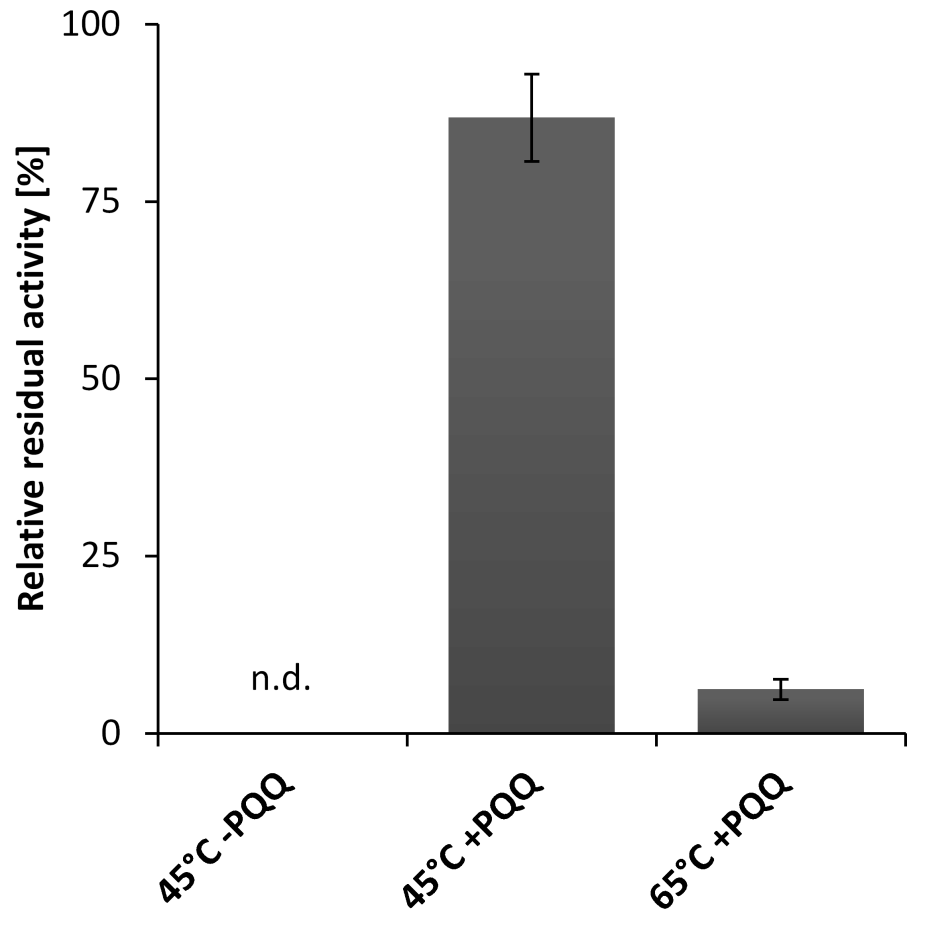
**

**Figure S2:** Relative residual activity of purified PedE wildtype protein upon incubation for 1 h at different temperatures with and without reconstitution with CaCl_2_ and PQQ prior to incubation. Data are presented as the mean of three biological replicates and error bars represent the corresponding standard deviations. Relative residual activities below detection limit are indicated (n.d.).

**Figure S3:** Specific activity of *E. coli* BL21(DE3) cells expressing PedE depending on cell density (OD_600_) used. Activities are mean of three independent measurements and error bars represent corresponding standard deviation.

**Table S1:** Primers used in this study. Variations are marked in bold and homologies to the plasmid pMW09 are marked in italic.

| Primer name | Sequence 5’ → 3’ | Annealing |
| --- | --- | --- |
| IHO01_PedE_307ATC_FWD | GTTCGACTACAAG**ATC**AAGAACGGCAAC | 65°C |
| IHO02_PedE_307ATC_REV | GTTGCCGTTCTT**GAT**CTTGTAGTCGAAC | 65°C |
| IHO03_PedE_307RHG_FWD | GTTCGACTACAAG**RHG**AAGAACGGCAAC | 65°C |
| IHO04_PedE_307RHG_REV | GTTGCCGTTCTT**CDY**CTTGTAGTCGAAC | 65°C |
| IHO05_PedE_310VMG_FWD | GACAAGAACGGC**VMG**GTGGTCAAGGCCAC | 65°C |
| IHO06_PedE_310VMG_REV | GTGGCCTTGACCAC**CKB**GCCGTTCTTGTC | 65°C |
| IHO07_PedE_310CGC_FWD | GACAAGAACGGC**CGC**GTGGTCAAGGCCAC | 65°C |
| IHO08_PedE_310CGC_REV | GTGGCCTTGACCAC**GCG**GCCGTTCTTGTC | 65°C |
| IHO09_PedE_408MHG_FWD | CTGGACCGAG**MHG**GTGAACTACAAG | 55°C |
| IHO10_PedE_408MHG_REV | CTTGTAGTTCAC**CDK**CTCGGTCCAG | 70°C |
| IHO11_PedE_408KRC_FWD | CTGGACCGAG**KRC**GTGAACTACAAG | 55°C |
| IHO12_PedE_408KRC_REV | CTTGTAGTTCAC**GYM**CTCGGTCCAG | 70°C |
| IHO17_PedE_410NCG_FWD | GACCGAGGAAGTG**NCG**TACAAGAAGG | 65°C |
| IHO18_PedE_410NCG_REV | CCTTCTTGTA**CGN**CACTTCCTCGGTC | 65°C |
| IHO19_PedE_410RAM_FWD | GACCGAGGAAGTG**RAM**TACAAGAAGG | 65°C |
| IHO20_PedE_410RAM_REV | CCTTCTTGTA**KTY**CACTTCCTCGGTC | 65°C |
| IHO25_PedE_91VAG_FWD | GACCTACAACCAC**VAG**CTGCCCGACAAC | 65°C |
| IHO26_PedE_91VAG_REV | GTTGTCGGGCAG**CTB**GTGGTTGTAGGTC | 65°C |
| IHO27_PedE_91GSC_FWD | GACCTACAACCAC**GSC**CTGCCCGACAAC | 65°C |
| IHO28_PedE_91GSC_REV | GTTGTCGGGCAG**GSC**GTGGTTGTAGGTC | 65°C |
| IHO61_PedE_352GMM_FWD | AGCCATATCGACCTG**GMM**ACCGGGCGCCCGGTG | 67°C |
| IHO62_PedE_352GMM_REV | CACCGGGCGCCCGGT**KKC**CAGGTCGATATGGCT | 55°C |
| IHO63_PedE_352CGC_FWD | AGCCATATCGACCTG**CGC**ACCGGGCGCCCGGTG | 63°C |
| IHO64_PedE_352CGC_REV | CACCGGGCGCCCGGT**GCG**CAGGTCGATATGGCT | 55°C |
| IHO65_PedE_352TGG_FWD | AGCCATATCGACCTG**TGG**ACCGGGCGCCCGGTG | 70°C |
| IHO66_PedE_352TGG_REV | CACCGGGCGCCCGGT**CCA**CAGGTCGATATGGCT | 70°C |
| MWH_QC26_PedE_Gibson_fwd | *CAATTCTTAAGAAGGAGATATACATATGACA*ATAAGATCGCTACCC | Varying |
| MWH_QC33_PedE_Gibson2_rev | *CGGTAGTCAATAAACCGGTA*AGCTTAGTGG | Varying |

**Table S2:** Overview of parameter optimization for the whole-cell activity assay using of *E. coli* BL21(DE3) cells expressing PedE. Specific activities (U gCDW^-1^) are the means of three individual measurements ± corresponding standard deviations. Activities below detection limit are indicated (n.d.).

* Depending on the desired pH, either 100 mM Tris (pH 8) or 100 mM phosphate (pH 5) buffer was used.

| pH* | DCPIP [µM] | PMS [µM] | Imidazol [mM] | PQQ [µM] | CaCl_2_ [mM] | Specific activity [U gCDW^-1^] |
| --- | --- | --- | --- | --- | --- | --- |
| 8 | 150 | 150 | - | - | - | n.d. |
| 8 | 150 | 150 | 25 | - | - | 28 ± 2 |
| 8 | 150 | 150 | - | 1 | - | n.d. |
| 8 | 150 | 150 | - | - | 1 | n.d. |
| 8 | 150 | 150 | - | 1 | 1 | \| 10 ± 5 \| \| --- \| |
| 8 | 150 | 150 | 25 | 1 | - | 37 ± 1 |
| 8 | 150 | 150 | 25 | - | 1 | 53 ± 2 |
| 5 | 150 | 150 | 25 | 1 | 1 | 76 ± 10 |
| 8 | 150 | 150 | 25 | 1 | 1 | 358 ± 13 |

**Table S3:** Biotransformations (500 µL) with 8 mM 2-phenylethanol using *E. coli* BL21(DE3) cells producing PedE (**A**) or 10 µg ml^-1^ of purified PedE enzyme (**B**) in DCPIP assay solution at 30°C. Control experiments were carried out identically as whole-cell biotransformations except for exchanging PedE producing cells with eGFP producing cells (**C1**), omitting DCPIP and PMS in the reaction buffer (**C2**), or using reaction buffer without addition of cells or enzyme (**C3**). Products that could not be detected are indicated (n.d.).

*Up to 0.06 mM phenylacetaldehyde were detected in all 0 h samples and control experiments, which originate from impurities of the utilized 2-phenylethanol substrate.

|  | **Time [h]** | **2-Phenylethanol [mM]** | **Phenylacetaldehyde [mM]** | **Phenylacetic acid [mM]** |
| --- | --- | --- | --- | --- |
| **A** | 0 | 7.3 ± 0.5 | < 0.07* | n.d. |
|  | 1 | 6.4 ± 0.2 | 1.3 ± 0.1 | n.d. |
|  | 24 | 2.9 ± 0.1 | 4.3 ± 0.1 | 0.4 ± 0.1 |
| **B** | 0 | 7.5 ± 0.1 | < 0.07* | n.d. |
|  | 1 | 3.7 ± 0.2 | 3.9 ± 0.1 | n.d. |
|  | 24 | 2.3 ± 0.2 | 4.5 ± 0.2 | 0.4 ± 0.1 |
| **C1** | 0 | 8.0 ± 0.1 | < 0.07* | n.d. |
|  | 1 | 8.0 ± 0.1 | < 0.07* | n.d. |
|  | 24 | 7.9 ± 0.1 | < 0.07* | n.d. |
| **C2** | 0 | 7.2 ± 0.4 | < 0.07* | n.d. |
|  | 1 | 7.6 ± 0.2 | < 0.07* | n.d. |
|  | 24 | 7.3 ± 0.2 | < 0.07* | n.d. |
| **C3** | 0 | 7.9 ± 0.1 | < 0.07* | n.d. |
|  | 1 | 7.8 ± 0.1 | < 0.07* | n.d. |
|  | 24 | 7.5 ± 0.1 | < 0.07* | n.d. |
